# What it Takes to Implement Population-Based Genomic Screening: A Multi-Site Qualitative Study of Implementation Determinants Across Health Systems

**DOI:** 10.21203/rs.3.rs-9418493/v1

**Published:** 2026-05-11

**Authors:** Caitlin G. Allen, Deborah Cragun, Miranda Hallquist, Adam H. Buchanan, Cason Whitcomb, Rebecca Bosch, Ingrid Wagner, Jarrod Marable, Kimberly Foss, Derek W. Craig, Mary-Louise Millett, Chanita Hughes Halbert, Nathaniel L. Baker, Megan C. Roberts

**Affiliations:** Wake Forest University School of Medicine; University of South Florida; Geisinger Health System; Geisinger Health System; University of North Carolina at Chapel Hill; University of North Carolina at Chapel Hill; Wake Forest University School of Medicine; Wake Forest University School of Medicine; University of North Carolina at Chapel Hill; University of Texas Health Science Center at Houston; University of Texas Health Science Center at Houston; University of Southern California; Medical University of South Carolina; University of North Carolina at Chapel Hill

**Keywords:** implementation science, population-based genomic screening, qualitative research, CFIR 2.0, health systems, implementation determinants, sustainment

## Abstract

**Background:**

Population-based genomic screening (PGS) holds promise for identifying individuals at elevated risk for hereditary conditions. However, the absence of implementation guidance limits the scalability and impact of these programs. This qualitative study aimed to identify facilitators and barriers to PGS implementation among healthcare settings at different phases of PGS implementation.

**Methods:**

We conducted qualitative interviews with implementation team members, including genetic counselors, physicians, information technology and informatics personnel, study coordinators, and institutional leaders across 10 PGS sites. Sites were categorized as pre-adoption, implementing, and sustaining. Interviews explored implementation experiences, from the decision to adopt a PGS program through the return of results. Transcripts were coded using a rapid qualitative analysis approach using the Consolidated Framework for Implementation Research (CFIR) 2.0. We completed a data matrix heat map to visualize differences in CFIR 2.0 determinants across phases of PGS implementation.

**Results:**

We completed 34 interviews across 10 PGS sites (four pre-adoption, four implementing, and two sustaining). We identified 13 key CFIR 2.0 determinants across sites. Pre-adoption sites reported barriers related to financing, low institutional priority, workflow complexity, and limited patient and provider genomic knowledge, with mixed views on compatibility and resources. Implementing sites cited complexity in building out workflows and infrastructure, gaps in downstream clinical capacity, and uneven provider engagement; external partnerships, supportive leadership, and strong digital infrastructure facilitated progress. Sustaining sites identified persistent knowledge gaps and resource strain despite mature workflows, but benefited from robust partnerships, strong relational connections with clinical champions and lab partners, and continued leadership engagement.

**Conclusions:**

PGS implementation is shaped by phase-specific inflection points and ongoing barriers and facilitators that evolve across implementation phases. Financing and institutional prioritization were critical to moving programs from pre-adoption to implementing, while partnerships and relational connections were essential for implementing and sustaining sites. Barriers identified across CFIR domains such as policy constraints, and ongoing knowledge assessments require adaptive strategies that are specific to the phase of implementation, rather than one-time solutions. These results underscore the importance of dynamic implementation strategies that evolve alongside the PGS program.

## INTRODUCTION

Population-based genomic screening (PGS) allows for early identification of genetic risk and initiation of risk management for a variety of inherited cancer and cardiovascular diseases. While 1–2% of the population has a pathogenic variant in a gene associated with hereditary breast and ovarian cancer syndrome, Lynch syndrome, or familial hypercholesterolemia, these conditions are currently underdiagnosed, leaving many individuals unaware of their risk.^1-7^ The number of organizations implementing PGS in the US has at least tripled since the National Academies of Science, Engineering, and Medicine’s Genomics Public Health Action Collaborative endorsed PGS in 2018.^3,6,8-10^ However, no clear guidelines or standardized implementation and evaluation strategies exist to support the rapid expansion of PGS programs in the US.^11,12^ While PGS holds tremendous potential to improve population health through early detection and tailored prevention, its successful integration into routine clinical workflows requires careful attention to the contextual and process-related factors that shape implementation.^8,13^

Implementation science is a vital bridge between the potential of genomic innovations and their practical application in healthcare settings.^8,12^ Implementation science methods provide a structured approach to uncovering how genomic information can be effectively integrated into care by identifying what works, for whom, and under what conditions. This is especially important in complex systems where implementation success depends on alignment across organizational priorities, clinician readiness, and patient engagement. Studies such as the Implementing Universal Lynch Syndrome Screening (IMPULSS) project have demonstrated the value of using implementation science frameworks to identify organizational determinants of success and variation in genomic program uptake across healthcare systems.^14^

Despite growing enthusiasm for PGS, national expert groups emphasize that the field is still in an early developmental phase, with substantial work needed to understand how PGS can be implemented responsibly and equitably at scale.^6,10,15^ Recent national reports highlight ongoing uncertainties around health system readiness for large-scale genomic screening, including challenges related to workforce capacity and readiness, result communication, and the infrastructure needed to support expanded screening.^6,11,15^ Yet, practice-based evidence describing how diverse health systems operationalize these multilevel challenges remains limited.^10,15^ This lack of applied knowledge poses a barrier to broader adoption and reinforces the need for detailed examinations of real-world PGS implementation processes across varied healthcare contexts. To address these gaps, we conducted a qualitative analysis and developed a data matrix heat map to assess implementation processes across ten health systems at various phases of implementing PGS in the US.

## METHODS

### Study Design & Participants

We used a multi-site qualitative study design, conducting interviews with health systems at various phases of PGS implementation. Sites were selected for representativeness across three different phases: pre-adoption, implementing, and sustaining. Pre-adoption sites were defined as evaluating the needs and potential fit of PGS but had not decided to adopt PGS. Implementing sites had made the adoption decision and were actively delivering PGS within their institution, integrating PGS into clinical workflows, and enrolling patients. Sustaining sites’ PGS programs were maintained or institutionalized within the organization’s routine operations and infrastructure.^16,17^ Interviewee roles included research coordinators, patient navigators, genetic counselors, provider champions, lab personnel, information technology and informatics personnel, marketing personnel, and business operations personnel. We applied a purposeful sampling strategy to capture insights from distinct roles required to implement PGS and variation among sites at different phases of PGS implementation.

### Data Collection

We completed semi-structured in-depth interviews to collect qualitative data on the implementation of PGS programs, using an interview guide informed by the Consolidated Framework for Implementation Research (CFIR) 2.0^18^ (**Supplementary Files**). CFIR 2.0 is a determinant framework designed to systematically identify contextual factors that influence implementation success. The interview guide included open-ended questions designed to explore team members’ beliefs about PGS and, for those at implementing or sustaining sites, their experiences across various phases of implementation ranging from the adoption decision through the return of results. We conducted interviews virtually via video conference software. Each session lasted 60 minutes on average, was audio-recorded, and transcribed verbatim. We completed member checking at the site level to reconcile any confusion or discrepant accounts, gain clarity across individual implementation team member interviews, and verify the accuracy of the qualitative analysis. Member checking interviews used a semi-structured focus group format with an interview guide informed by our learnings from the individual implementation team member interviews (**Supplementary Files**).

### Data Analysis

We completed rapid qualitative analysis using an interview summary template guided by the CFIR 2.0 constructs. The primary coder drafted each interview summary, extracting CFIR 2.0 constructs from the interviews. A secondary coder independently completed coding, after which any discrepancies in the interview summaries were addressed and resolved in a two-tiered process. First, primary and secondary coders met and reviewed each site’s interview transcript summaries. During this process, barriers, facilitators, and implementation strategies for PGS program implementation were extracted and organized for cross-site and cross-phase analysis. Primary and secondary coders then conducted member checking with implementation team members from the corresponding site. Barriers and facilitators that arose during member checking were incorporated.

At the conclusion of qualitative analysis, we completed a data matrix heat map to compare barriers and facilitators across sites.^19-22^ One team member summarized the coded data to create a data matrix for each site. To do this, we listed CFIR 2.0 factors in columns and included relevant quotes and summaries in the respective cells so that responses from each interview were represented across a single row within the data matrix for the respective organizational unit. One team member applied color-coded^23,24^ valence to the summaries for each construct for each stakeholder independently and then a second team member reviewed to resolve discrepancies. Valences were as follows: facilitator (positive), mixed-positive (positive mention more than 50% of the time), mixed (50/50 mix of positive and negative), mixed negative (> 50% negative), barrier (negative), not mentioned.^25^ Coders resolved discrepancies through discussion and review of the original transcripts to help contextualize and ensure accuracy of summaries. We then combined individual-level valences within the same site to create a site-level valence. After completing initial data matrices, we combined site-level summary valences from the data matrix heat maps of each site into a single data matrix for comparisons. We grouped sites into pre-adoption, implementing, and sustaining to visualize barriers and facilitators across phases of PGS implementation, assessing for any similarities and differences.

## RESULTS

### Site and Participant Demographics

We completed interviews with 34 participants from 10 institutions at different phases of PGS implementation: pre-adoption (n = 4), implementing (n = 4), and sustaining (n = 2). Member checking was completed with nine of the 10 sites. Implementing and sustaining sites had enrolled between 300–300,000 individuals in PGS with a program age of one year to greater than 10 years (Table 1).

Interviewees included institutional leaders (n = 7, 20.6%), principal investigators (n = 7, 20.6%), genetic counselors (n = 5, 14.7%), study coordinators/managers (n = 4, 11.8%), primary care clinicians (n = 3, 8.8%), IT personnel (n = 2, 6%), marketing personnel (n = 2, 6%), institutional lab personnel (n = 1, 3%), and other personnel (n = 3, 8.8%). Interviewee ages ranged from 25 to 68 years, with a median of 47. Most interviewees identified as female (n = 25, 73.5%), White (n = 23, 67.6%), and non-Hispanic (n = 31, 91%) (Table 2).

### Pre-Adoption Site Themes

#### Pre-Adoption Site Barriers

**Figure 1:**
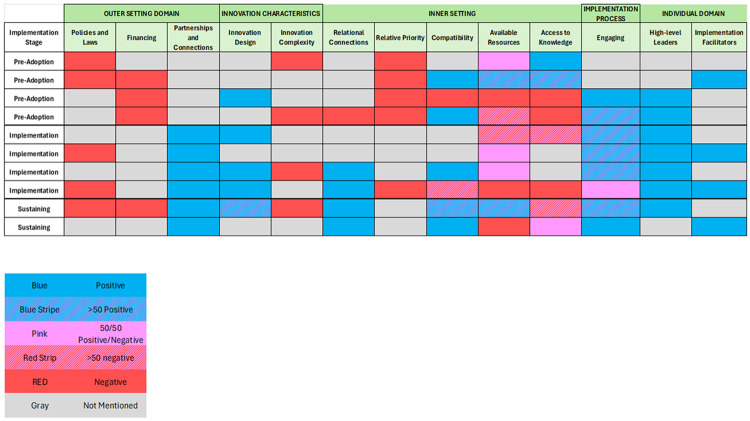
Data Heat Map Matrix

Pre-adoption sites were exploring the option and potential fit of PGS but had not yet made the decision to adopt PGS (Fig. 1, Table 3). Commonly mentioned anticipated barriers among pre-adoption sites included concerns about policies and laws that would impact PGS adoption, financing uncertainty and need to rely on external resources, complexity of PGS implementation process, lack of relational connections required for PGS implementation, and competing priorities (relative priority). **Financing** shaped whether PGS could move forward, with the majority of sites describing uncertainty about long-term program funding as a key challenge. Programs at this phase indicated that they would need to depend on external or research-based funding, which was considered insufficient for adoption and generated hesitation among operational leaders. **Relative priority** was also a major barrier among sites considering PGS, as PGS was described as important but not urgent, especially with many competing demands and highly visible operational goals. Practical constraints further reinforced low prioritization (e.g., informatics teams required to build workflows and providing genomic results). For example, “*There's always competing priorities…The EPIC engineers and people who can make changes and do things in EPIC…they're always busy*” (Site 2, IT Personnel).

#### Pre-Adoption Site Mixed

Compatibility of PGS with existing workflows, available resources (e.g., insufficient staffing, constrained information technology and informatics capacity, existing digital tools), and access to knowledge were highly mixed across pre-adoption sites. Many individuals felt that their organization could in principle support PGS but were concerned about **compatibility.** For example, pre-adoption sites had already implemented some initiatives that were aligned with PGS (e.g., digitally driven patient communication and recruitment processes), but there was concern that PGS might disrupt clinical workflows, require new processes, or introduce responsibilities they were unable to manage. One site stated, *“Sometimes we find that providers [feel] it's going to disrupt their flow…a big change…it opens up a can of worms…on board involvement from staff…is probably one of those factors that kind of slows it down…So if you don’t have them on board…you’re not gonna get anywhere"* (Site 1, Other Personnel). **Available resources** were also mixed, with some organizations reporting strong research infrastructure, existing partnerships, or digital tools that could support genomic screening initiatives. For example, one interviewee noted, *"We actually have a pretty good infrastructure in place because of our research studies…expanding some of the initiatives we've done that are risk-based now but make them more population-based…So I feel like we’ve actually got some nice…approaches that would certainly [be used] at the beginning part of that workflow"* (Site 2, Principal Investigator). However, despite these existing resources, sites remained concerned about their capacity to manage downstream clinical workflows necessary for large-scale genomic screening programs. **Access to knowledge** was mixed and often insufficient at pre-adoption sites among both clinicians and patients. Primary care providers were identified as a group with the greatest need for additional education, but lack of patient knowledge was also a concern (e.g., patient confusion about genetics, varying levels of health literacy, and implications of receiving genetic results).

#### Pre-Adoption Site Facilitators

Anticipated facilitators among pre-adoption sites included clearly defined workflows for PGS implementation (innovation design), engagement of leadership and clinical champions, high-level leader willingness to prioritize PGS, and implementation facilitators who had expertise in PGS implementation. **Engagement** among pre-adoption sites included planning meetings among champions, navigators, and cross-department collaborators to demonstrate early progress toward PGS. All pre-adoption sites described the importance of having diverse and engaged constituents who could bridge departments, navigate governance structures, and maintain momentum necessary to support a PGS program. For example, *“Without a navigator… someone who can help you get through all the levels of governance, it would be almost impossible”* (Site 2, Principal Investigator). **High-level leaders** were also considered a strong, crosscutting facilitator whose support would help demonstrate that PGS could become a feasible institutional initiative and a critical anchor for progress in alignment with broader organizational values and goals.

#### Implementing Site Themes

##### Implementing Site Barriers

Implementing sites were actively delivering PGS within their institution, integrating PGS recruitment into clinical workflows, and enrolling patients. Commonly mentioned barriers included institutional policies, innovation complexity requiring technical builds and integration of multiple IT systems, lack of available resources, and insufficient access to knowledge. **Availability of resources** was considered a barrier across all implementing sites. The primary concern about available resources was the clinical and operational capacity required to support downstream follow-up for those identified as high-risk through PGS. These challenges were not just among providers, but also staff, schedulers, and genetic counselors who are required to triage and manage downstream management. For example, *“He’s [site gastroenterologist] already struggling… to find time in his really busy clinic to add them on [PGS positive patients]”* (Site 5, Principal Investigator). Another concern about available resources was the ability to continuously support PGS workflows after initial implementation. While many sites reported strong pre-existing infrastructure, the implementation process often uncovered gaps in resources (e.g., IT teams, Electronic Health Record [EHR] teams) that would be required to maintain genomics workflows long term.

#### Implementing Site Mixed Themes

Compatibility and engaging were highly mixed among implementing sites. Many sites described strong **compatibility** or alignment between PGS and existing digital infrastructures that allowed for easy invitations and streamlined communication for recruitment, consent, and results delivery. Challenges emerged with the compatibility of workflows that required coordination across multiple clinical teams or returning results (e.g., results reports were not easily integrated into the EHR). One site shared, *“[Lab] reports have a lot of information, but they’re not something patients or primary care providers can really look at and see what to do next… developing a system to address that was a big hurdle”* (Site 7, Principal Investigator). While many implementing sites indicated strong leadership endorsement and effective system-wide marketing efforts to recruit PGS participants, there was mixed **engagement** of clinicians. One site indicated that despite institutional leadership endorsement and encouragement for clinician engagement, initial assumptions about clinician engagement were overly optimistic.

#### Implementing Site Facilitators

Facilitators among implementing sites included: high-quality external partnerships and connections, high-quality innovation design, sponsorship and hands-on support from high-level leaders, and strong relational connections that allow for high-quality communication across the site. **Partnerships and connections** were considered a facilitator among all implementing sites. These sites relied heavily on external collaborators, including industry partners and genetic testing companies, to support program start-up and success (e.g., IRB material templates, recruitment content, clinical documentation, operational templates). This approach helped reduce institutional burden and accelerated implementation. **Innovation design** was also a major facilitator among implementing sites. Automation of recruitment through digital tools and EHR-integrated workflows made it possible to rapidly expand and enroll patients quickly. For example, *“The fact that this is all integrated and we can literally send out batches of 20,000 invitations… to make it this really seamless process to invite people and then… you can join anywhere you are from your phone, so it’s really an easy consent process”* (Site 5, Principal Investigator). Finally, **high-level leaders** played a critical role in supporting PGS across all implementing sites. Strong endorsement from institutional leadership (e.g., department chairs, system-level executives, and clinical leaders) that actively championed genomic screening helped provide legitimacy, resources, and strategic alignment for PGS implementation. For example, *“One of the reasons I came to this institution was that I had met the Chair of Pathology… her vision and my vision overlapped… she had already worked with high-level leadership and other department chairs to socialize it and get buy-in for a pilot”* (Site 7, Principal Investigator).

#### Sustaining Site Themes

##### Sustaining Site Barriers

Sustaining sites have established ongoing PGS programs and demonstrate long-term integration of PGS into their clinical workflows. Barriers among sustaining sites included unclear future financing, complexity of sustaining the intervention, and ensuring ongoing access to knowledge. **Access to knowledge** was a barrier for both sustaining sites. Both noted ongoing gaps in provider and patient understanding related to genomic screening processes, results interpretation, and appropriate clinical follow-up. Despite established workflows and prior exposure to genomics initiatives, sites indicated that many clinicians required continued education and periodic reinforcement to maintain familiarity with ordering pathways, referral processes, and management of positive findings. For example, *"[the challenge] was teaching the ordering docs the appropriateness of when the testing should be”* (Site 9, Institutional Leadership). Sustaining sites also reported ongoing patient challenges in access to knowledge and understanding of the purpose of PGS, the meaning of results, and implications for their care.

#### Sustaining Site Mixed Themes

Innovation design and available resources were highly mixed among sustaining sites. PGS workflows were considered to be well-established and included features that supported efficient return of results; however, some reported reliance on clinician memory for eligibility-based enrollment. **Available resources** reflect the challenges in the long-term maintenance of PGS programs. Strong existing infrastructures (e.g., communications teams, experienced genetics personnel, and EHR-embedded tools) allowed for continued program operations beyond initial implementation. However, sites faced resource-related barriers during sustainment, particularly around the downstream follow-up capacity and added workflows for genetic teams (e.g., continued demand on personnel to coordinate genetic counseling and support follow-up care). One site stated, *“The first thing is physician acceptance… the second is an easy way to order the test… and the third is to have a team of genetic counselors, who can provide detailed information to the patient about their test results”* (Site 9, Primary Care Clinician).

#### Sustaining Site Facilitators

Facilitators among sustaining sites included leveraging external expertise through strong partnerships and connections, collaborative relational connections, PGS workflow compatibility, engaging knowledgeable staff, strong recruitment strategies, and high-level leaders support. **Partnerships and connections** played a vital role among sustaining sites, with sites leveraging external expertise to strengthen educational materials and provide specialized input that supplemented internal capacity. This allowed for refinement of workflows and continual updates as testing panels are updated. **Relational connections** were also important facilitators for sustaining sites, with well-established internal communication channels and collaborative relationships across departments that facilitated continued coordination, follow-up and program reach. These connections allowed teams to manage ongoing demands and troubleshoot more efficiently. For example, *“It is very helpful when their PCPs are looped in with their care… the more patients are looped in with their PCP and the PCP is aware of this new condition or new result, the more likely they are to follow up with those specialists and actually go through with the recommended care”* (Site 9, Genetic Counselor). Ensuring **compatibility** with workflows and organizational systems helped ensure that PGS was integrated and minimized maintenance burden over time (e.g., automated reminders or embedded outreach campaigns). Finally, **engaging** leadership helped ensure continual endorsement of PGS initiatives, communication between teams to support system-wide outreach, and provider engagement with sample collection and follow-up.

## Discussion

This multi-site qualitative study identified determinants that influence PGS implementation across pre-adoption, implementing, and sustaining phases. We identified phase-specific determinants and cross-cutting determinants, underscoring the importance of phase-tailored implementation strategies when implementing and scaling PGS.

A set of barriers and facilitators persisted across phases of implementation, with nuanced differences in how it functioned across implementation phase. For example, complexity was considered a barrier across pre-adoption, implementing, and sustaining. For pre-adoption sites, complexity was considered a barrier due to concerns about scalability, downstream consequences, and uncertainty about managing genomic findings. This is consistent with prior work demonstrating that perceived complexity can inhibit early adoption of genomic innovations.^11^ These concerns shifted to technical and infrastructure complexities that had to be overcome (e.g., EHR builds, multi-system integration, and coordination) among implementing sites, which aligns with studies highlighting the operational burden of integrating genomics into clinical systems.^14,26^ In sustaining, complexity further shifted to a large-scale operational burden (e.g., processing large quantities of individuals with positive PGS results, follow-up capacity), reflecting that implementation complexity often results from capacity strain over time.^6,27^

Similar to barriers, a core set of facilitators (e.g., high-level leaders, partnerships and connections, innovation design), persisted across implementation phases, though nuanced differences were also noted. Together, these facilitators enabled programs to move from exploration of PGS to execution and long-term sustainment. For example, high-level leaders were a facilitator across all phases, but their role shifted. During pre-adoption, high-level leaders provided legitimacy and strategic alignment to demonstrate that PGS was an institutional priority. During implementing, this shifted to focus on execution alongside implementation staff and problem-solving implementation challenges. In sustaining, high-level leaders ensured continued endorsement of the PGS program. These findings are consistent with prior implementation research demonstrating that leadership engagement is a cross-cutting determinant of success, though its functional role may shift from strategic endorsement to operational support and maintenance.

Other determinants (e.g., compatibility, available resources, access to knowledge), were present across all phases, but shifted from barrier to facilitator. For example, in pre-adoption and implementing, compatibility was often mixed or uncertain. Concerns included workflow disruption, challenges with EHR integration, and clinician preparedness for downstream clinical management of high-risk individuals. These findings align with prior literature indicating that misalignment between genomic innovations and existing workflows can impede adoption.^8,10^ In contrast, among sustaining sites, compatibility was considered a facilitator. By embedding PGS into existing clinical systems through automated outreach for recruitment to the program and return of negative results, EHR infrastructure (e.g., documentation templates, alerts), sustaining sites reduced maintenance burden.

Taken together, these findings suggest that PGS implementation is likely shaped by a core set of determinants that persist across phases, but the functions shift over time. Thus, it is critical to address these core determinants when implementing PGS. This challenges the implicit assumption/paradigm that determinant identification must be repeated in each new context. Rather, our findings indicate that these core determinants are consistently present and therefore do not require continual re-identification. Instead, the key task for implementation is understanding how these determinants evolve in how they manifest within a specific context. This reframing shifts the focus of implementation efforts from identifying *what* determinants exist to understanding *how* they function and *how* to address them dynamically across phases (e.g., leveraging facilitators and overcoming barriers).

This pattern for continual reassessment of determinants and adaptation of implementation strategies aligns with and extends the Exploration, Preparation, Implementation, Sustainment (EPIS) framework that predicts shifting salience of determinants across phases.^28,29^ According to EPIS, in pre-adoption (i.e., during exploration and preparation phases), determinants manifest as perceptions and anticipatory concerns. These shift to workflow and infrastructure challenges during implementation, and reappear as issues related to capacity, fatigue, and system constraints during sustainment. Our results underscore the need to map core determinants to phase-specific implementation strategies.^30-32^ For example, early-phase strategies for PGS adoption should focus on reducing uncertainty, clarifying the relative advantage of PGS, and establishing organizational fit between existing workflows and PGS workflows.

Implementation-phase strategies should prioritize workflow integration, infrastructure development to support PGS, and clinician engagement. In sustaining sites, strategies should emphasize automation or streamlining of processes (e.g., recruitment and return of results, embedding PGS workflows into the EHR, reduction of clinician burden, and strengthening the follow-up care pathways) to support sustainment. This phased approach is consistent with prior work emphasizing the importance of tailoring implementation strategies to context and stage of implementation and highlights the need for adaptive strategy selection over time rather than one-time implementation planning.^33-35^

Beyond the core determinants that evolve across implementation phases, there were notable phase-specific determinants. Among pre-adoption sites, financing and relative priority were two salient barriers. These sites consistently described financing as uncertain, fragile and dependent on external research funds. Without sustainable reimbursement pathways, leaders at pre-adoption sites were hesitant to adopt PGS, which highlights a potential mismatch between enthusiasm for PGS and financial viability. Financing was not raised as a barrier among actively implementing or sustaining sites (though future sustained funding was a concern among one sustaining site). This suggests that once programs launch, funding concerns stabilize or perhaps become secondary to other barriers such as operational challenges. These findings align with prior studies demonstrating that early PGS programs are frequently supported by pilot funding and lack clear long-term financial models.^6,10^ The relative priority of PGS was also unique to pre-adoption sites. Although PGS was considered important among leaders, it was not seen as critical. Other institutional demands and priorities were a limiting factor to pursue PGS, especially considering the perceived investment of IT and EHR resources. These findings suggest that early-phase implementation strategies should explicitly target financing and prioritization concerns, for example by clarifying long-term funding pathways, articulating return on investment, and increasing the prioritization of PGS in comparison with other institutional priorities.^10^ Once implementation is underway, however, strategies may need to shift toward addressing enduring operational determinants rather than revisiting adoption-stage concerns.^36^

This study has several limitations. Although we included 10 sites across multiple phases of implementation, the number of sites within each phase was limited, which may affect generalizability of phase-specific findings or may have limited the variety of barriers identified. Interviewees were primarily involved in PGS implementation and may represent early adopters, introducing potential selection bias. Findings are also subject to recall and social desirability bias. While CFIR 2.0 provided a structured approach to data collection and analysis, its use may have constrained identification of determinants outside of the framework. Additionally, implementation phases (pre-adoption, implementing, and sustaining) were categorized for analytic purposes but in practice these stages may be non-linear or may be overlapping.

In conclusion, this multi-site qualitative study identified both enduring and phase-specific determinants shaping PGS implementation across health systems and demonstrates that implementation determinants are both predictable and dynamic across phases. A core set of determinants consistently influenced PGS implementation, suggesting that many challenges—such as competing priorities or the need for leadership engagement—can be anticipated a priori, even prior to pre-adoption needs assessments. However, while these determinants persisted across phases, their functions and operational implications evolved over time; for example, leadership support was critical throughout implementation, yet the specific actions and forms of engagement required of leaders varied by phase. In contrast, other determinants were salient only at particular phases and appeared to shape the transition to subsequent stages of implementation. Together, these findings suggest that effective PGS implementation depends not only on identifying predictable determinants, but also on recognizing their dynamic, phase-contingent expression and aligning implementation strategies accordingly. By reframing determinants as both predictable and dynamically enacted rather than static, this work advances implementation science and underscores the importance of adaptive, phase-specific strategies to support the scalable and sustainable integration of PGS in health systems.

## Supplementary Material

This is a list of supplementary files associated with this preprint. Click to download.

• SupplementalMaterials.docx

## Figures and Tables

**Table 1:** Description of Sites

Site Number	Implementation Stage	Number of Interviewees	Number Enrolled in PGS at time of Interview	Regional Geographic Distribution	Program Lifespan (Years)
1	Pre-Adoption	4	N/A	Southeast US	N/A
2	Pre-Adoption	4	N/A	Western US	N/A
3	Pre-Adoption	2	N/A	Southeast US	N/A
4	Pre-Adoption	4	N/A	Northeast US	N/A
5	Implementation	2	35,611	Midwest US	1
6	Implementation	3	29,205	Midwest US	3
7	Implementation	3	323	Northeast US	6
8	Implementation	4	6,500	Southeast US	4
9	Sustainment	6	300,000	Northeast US	11
10	Sustainment	2	17,817	Midwest US	8

**Table 2:** Participant Demographics Information (n = 34)

Interviewee Role	n	%
Institutional Leadership	7	20.6
Principal Investigators	7	20.6
Genetic Counselor	5	14.7
Study/Program Coordinator/Manager	4	11.8
Primary Care Providers	3	8.8
Other Personnel	3	8.8
IT Personnel	2	6
Marketing Personnel	2	6
Provider Assistants (Medical Assistants, CNAs, etc.)	0	0
Institutional Lab Personnel	1	3
**Gender**		
Female	25	73.5
Male	9	26.5
**Age**	Median: 47	Standard Deviation: 11.70
**Race**		
White	23	67.6
Asian	8	23.5
Black or African American	2	5.8
More Than One Race	1	2.9
Native Hawaiian or Pacific Islander	0	0
American Indian/Alaskan Native	0	0
**Ethnicity**		
Non-Hispanic or Latino	31	91
Hispanic or Latino	3	8.8

**Table 3:** Summary of Facilitators and Barriers for Sites Across Implementation Stage

CFIR/HE Construct	Study Definition	Pre-Adoption		Implementation		Sustainment	
		Summary	Quote	Summary	Quote	Summary	Quote
Policies and Laws	The degree to which legislation, regulations, professional group guidelines and recommendations, or accreditation standards support implementation and/or delivery of PGS	Insurance coverage and cost-coding infrastructure	“We need to do campaigns among payers… to make sure all the payers update their coding system and understand the service can be covered. Even though the policy is there, it doesn’t mean implementation will go smoothly.” (Site 3, Principal Investigator)	IRB approvals	“This workflow needs to go through IRB, you know and. .. And it's not that those checks and balances were bad, but it kind of it delayed at times that our ability to kind of maybe move a little bit more quickly. .. You just have more players and basically more checkpoints throughout the way and I think that was one of the biggest things.” (Site 6, Principal Investigator)	Implementation and adoption timeline disruption	“[Lab] is do underlying pass… it’s validated, can’t act o you’re foun have a var has to be validated.” Institution Leadership
Financing	The degree to which funding from external entities is available to implement and/or deliver PGS (including NIH/federal funding)	Uncertain long-term financing and reliance on external funds	“Right now it’s research funding… until we can figure out how to get insurance to pay for it.” (Site 1, Study Manager)	N/A	N/A	Unclear future funding	“If there w funding so would hav a sea chan shift in the things go r now… if th an adequa funding so this would adaptable Institution Leadership
Partnerships and Connections	The degree to which the inner setting (e.g., PGS program + site) is networked with external entities, including referral networks, academic affiliations, and professional organization networks	N/A	N/A	Heavy reliance on external partners	“[Our partner (Lab)] came to the table with a ton of resources as far as the IRB protocol, clinical materials, recruitment materials, etc.” (Site 5, Principal Investigator)	Leveraging external expertise and collaboration	"We've wo with a gen counselor external ge counselor consultativ to help bui some of o patient fac materials." Program Manager)
Innovation Design	The degree to which PGS is well designed and packaged, including how it is assembled, bundled, and presented	Clear educational materials to build PGS trust	“All the information is clearly laid out so patients have something they can refer back to (p.11) … For our patient population, having a strong network of trust and patients being able to consent willingly is important… there’s a lot of mistrust and confusion… we want to make sure this is a safe space to proceed (p.7)” (Site 1, Genetic Counselor)	Digital workflow tools (e.g. MyChart) simplified recruitment, consent, and results return.	“The fact that this is all integrated and we can literally send out batches of 20,000 invitations… to make it this really seamless process to invite people and then… you can join anywhere you are from your phone, so it’s really an easy consent process.” (Site 5, Principal Investigator)	Mixed; Facilitators: design features support accurate results and reduce participation burden; Barriers: reliance on provider memory for eligibility-based enrollment, limited accommodation of patient needs (e.g., privacy concerns, lack of translated materials)	"I think tha have a goo system…h the funnel positives directly to team to re and then d is really he is helpful t that first re and then a review by m because… confident result bein positive." (Genetic Counselor “We have Hispanic patients… have a lot distrust… n offering an opportunit trace your genetics… going to be some data my French help me tr quickly int Spanish… translation equipment clunky… if had a tran explanatio could depl number of it would be helpful.” (S Primary Ca Provider)
Innovation Complexity	The degree to which the PGS is complicated, which may be reflected by its scope and/or the nature and number of connections and steps	Complexity and scalability concerns	"There's this concern…that you're sort of opening up Pandora's box and you're…finding out things that you don't have the answers for yet." (Site 3, Other Personnel)	Integration of multiple IT systems	“The hardest part was getting everything set up accurately… the technical build was the most time consuming and elaborate." (Site 8, Program Manager)	Batch result returns create processing delays and staff burden	“One of the challenges that we ge sequences large batch there’s not trickle of r return. It’s bolus of, ‘G now we ha 1,700 resu return,’ an want to ge into patien hands as q as we can, then we’ll b lull waiting next batch always bee challenge. Program Manager)
Relational Connections	The degree to which there are high quality formal and informal relationships, networks, and teams within and across inner setting boundaries (e.g., structural professional).	Siloed leadership across key groups (cancer center, PCP, IT)	"It would just need some new bridges between the Cancer Center, primary care, and cardiovascular leadership…getting everyone on the same page, talking about the same process, and making sure it works for everybody's sector or area." (Site 2, Principal Investigator)	Internal communications and marketing teams support awareness and recruitment	"The most helpful has been the advertising campaign through both [Site] communications as well as a person that our study hired to work with the [Site] communications team…to really make sure that we're spreading the word across the state." (Site 8, Principal Investigator)	Collaborative relationships and communication infrastructure support coordination, follow-up, and program reach.	“It is very h when their are looped their care… more patie looped in w their PCP a PCP is aw this new c or new res more likely are to follo with those specialists actually go through w recommen care.” (Site Genetic Counselor
Relative Priority	The degree to which there are high quality formal and informal relationships, networks, and teams within and across inner setting (PGS program) boundaries (e.g., structural professional)	PGS not prioritized due to competing priorities	"The main challenge is really prioritizing the idea of a population-based genomic screening program [it] has never been prioritized.” (Site 2, Principal Investigator) "There's always competing priorities…The EPIC engineers and people who can make changes and do things in EPIC…they're always busy." (Site 2, IT Personnel)	Difficulty obtaining physician buy-in amid the COVID-19 pandemic and competing priorities	“We tried to do things but it was just it didn't go really anywhere and. .. So that was problematic. I think we were working with family medicine doctors. And I think that they don't have the and I don't know the better system I, but they don't have a lot of time. So I, I I and then COVID happened. So COVID happened just as we were getting up and running.” (Site 7, Principal Investigator)	N/A	N/A
Compatibility	The degree to which PGS fits with the workflow, systems, or processes	Mixed positive; Existing workflows and IT systems support PGS, but providers worry PGS will disrupt workflows and that they are unprepared for follow-up care.	“Most helpful is something that doesn’t take a lot of extra effort… asynchronous communication… chatbots, things like that." (Site 2, Institutional Leadership) "Sometimes we find that providers [feel] it's going to disrupt their flow…a big change…it opens up a can of worms…on board involvement from staff…is probably one of those factors that kind of slows it down" (Site 1, Other Personnel)	Mixed EHR compatibility: workflows facilitated implementation, but EHR integration challenges created barriers	“When results come in, they don't go to providers, they come to the research team and I think that's important because the providers wouldn't handle it in a programmatic way like we do." (Site 8, Principal Investigator) “[Lab] reports have a lot of information, but they’re not something patients or primary care providers can really look at and see what to do next… developing a system to address that was a big hurdle.” (Site 7, Principal Investigator)	Positive or mixed positive compatibility for sustaining sites; workflow burden and limited EHR integration created barriers, while streamlined workflows, EHR tools (e.g., smart phrases, alerts), and prior system experience facilitated implementation	"We had no alerts ever EPIC/EMR 'Hey, this p would be a candidate this to.' So counts on providers k about the and electin offer that sampling t patient." (S Primary Ca Provider) “We have a phrase tha type into th notes so w on the gen lab end tha talked abo works real it’s easy fo to do, hope easy to rem and it’s ea us to double-che notes for t word.” (Sit Genetic Counselor
Available Resources	The degree to which resources are available to implement and deliver PGS (e.g., funding, space, materials and equipment)	Mixed — varies across sites; Barriers: insufficient staffing, limited follow-up capacity, and constrained IT/EHR resources; Facilitators: existing infrastructure, personnel, and digital tools supported communication and clinical workflows	"As of right now…anybody who has a positive test…gets sent to our genetic counselor… As our program grows, I don't know if that's going to be feasible because there's only one of her." (Site 1, Study Manager) "We actually have a pretty good infrastructure in place because of our research studies…expanding some of the initiatives we've done that are risk-based now, but make them more population-based." (Site 2, Principal Investigator)	Mixed; Barriers: limited staffing and downstream clinical capacity; Facilitators: existing infrastructure, marketing capacity, and funding	“He’s already struggling… to find time in his really busy clinic to add them on [PGS positive patients].” (Site 5, Principal Investigator)	Mixed; Barriers: limited follow-up capacity and added workload for providers and genetics teams; Facilitators: GC availability, PCP buy-in, and integrated workflows supported follow-up and sustainability.	“A lot of pr were supp but it was them to ad into their a busy work when they meeting w patients.” (Other Pers “The first t physician acceptanc second is way to ord test… and is to have of genetic counselors can provid detailed informatio patient abo test result 9, Primary Provider)
Access to Knowledge	The degree to which guidance and/or training is accessible to implement and deliver PGS	Mixed; Barriers: limited patient understanding and provider unfamiliarity with PGS workflows; Facilitators: provider education, familiarity with PGS workflows and testing, and prior technical knowledge	“[PCPs] don’t know how to order the test… don’t know where in the EHR to send a referral… don’t know how long it takes… the lack of selfefficacy and knowledge about the process was very consistent.” (Site 2, Principal Investigator)	Insufficient knowledge, training, and lack of standardized onboarding process	“There’s misunderstanding about what it is… what it isn’t… misunderstanding about what [Site] Genomic Health is compared to referring someone for clinical genetic testing and evaluation.” (Site 5, Principal Investigator)	Mixed negative; Barriers: limited provider and patient understanding, ongoing needs for education and workflow training; Facilitators: clinician buy-in and internal staff awareness	"[the challe was teach ordering d appropriat of when th testing sho (Site 9, Institution Leadership "Many of t who've bee involved sa benefit…pa who'd rece results and bought int another pi informatio can help m for my patient…cl said it's lik colonosco like a vacc something that I woul for their he (Site 9, Pro Manager)
Engaging	The degree to which attract and encourage participation in implementation and/or PGS	Mixed-positive; Facilitators: leadership buy-in, champions, and incentives; Barrier: PCP leadership burden concerns	“Without a navigator… someone who can help you get through all the levels of governance, it would be almost impossible.” (Site 2, Principal Investigator)	Mixed-positive; Facilitators: strong leadership support, champions, and marketing; Barriers: inconsistent provider buy-in, knowledge gaps, and operational challenges	"There's a lot of institutional leadership endorsement… leadership encouragement for collaborating sites and more physician engagement…” (Site 8, Principal Investigator) “I overestimated enthusiasm from providers…people weren’t gonna do it because it was not a guideline or they didn’t feel like they understood it enough.” (Site 7, Principal Investigator)	Mixed-positive; Facilitators: leadership buy-in, clinician engagement, knowledgeable staff, strong communication and recruitment strategies; Barriers: provider education needs and trust-building around genetics	"The team recently implement some MyC campaigns remind peo provide the sample…k it at the fo of their mi [Site GS] corporate communic team…wor with us to those toge (Site 9, Pro Manager)
High Level Leaders	Individuals with high level of authority, including key decision-makers, executive leaders, or directors	Leadership openness, prioritizes quality improvement and efficiency	“He’s very willing to hear out different people from different sources… he’s definitely open to considering all angles and perspectives.” (Site 1, Genetic Counselor)	Active senior-leader sponsorship and hands-on leadership driving program adoption and implementation	“One of the reasons I came to this institution was that I had met the Chair of Pathology… her vision and my vision overlapped… she had already worked with high-level leadership and other department chairs to socialize it and get buy-in for a pilot.” (Site 7, Principal Investigator)	Leadership-appointed genetics expert	“The execu leadership system as the…Geno Institute…o an MD gen with a bac in informa the other i geneticist now the C Scientific O they were placed. Th not people simply wit MBA who to be there 9, Institutio Leadership
Implementation Facilitators	Individuals with subject matter expertise who assist, coach, or support implementation	Internal expertise and leadership familiarity	"I have a hybrid role where I support research and operational activities. One of my areas of responsibility is genetics. So I'd say our team knows quite a lot about it and our leadership is familiar." (Site 4, Principal Investigator)	Dedicated roles and subject-matter expertise	"The team involved a genetic counselor who worked as a variant analyst and had commercial clinical experience to be sort of my clinical partner, to bounce things off of and get some things done." (Site 7, Principal Investigator)	Project management coordination and support	"Having a manager w extremely beneficial. Coordinati between departmen making su on schedu having kno of a past p was helpfu 10, Other Personnel)

## Abbreviations

CFIR: Consolidated Framework for Implementation Research
EHR: Electronic Health Record
EPIS: Exploration, Preparation, Implementation, Sustainment
IMPULSS: Implementing Universal Lynch Syndrome Screening
PGS: Population-based genomic screening
